# ﻿Four genera of the subfamily Opiinae Blanchard (Hymenoptera, Braconidae) new for Japan, with the description of two new species

**DOI:** 10.3897/zookeys.1206.125662

**Published:** 2024-07-08

**Authors:** Yunjong Han, Cornelis van Achterberg, Hyojoong Kim

**Affiliations:** 1 Animal Systematics Laboratory, Department of Biological Science, Kunsan National University, Gunsan, 54150, Republic of Korea Kunsan National University Gunsan Republic of Korea; 2 Naturalis Biodiversity Center, P.O. 9517, 2300 RA Leiden, Netherlands Naturalis Biodiversity Center Leiden Netherlands

**Keywords:** *Areotetes*, identification, *Indiopius*, Japan, key, *Neopius*, new record, new species, parasitoid, *Sternaulopius*

## Abstract

Four genera are reported for the first time from Japan (*Areotetes* van Achterberg & Li, 2013, *Indiopius* Fischer, 1966, *Neopius* Gahan, 1917 and *Sternaulopius* Fischer, 1965), and keys are provided for the species of these genera. Two new species are described and illustrated: *Areotetesconvergens***sp. nov.** and *Sternaulopiusmaculiferus***sp. nov.**

## ﻿Introduction

The large and cosmopolitan subfamily Opiinae Blanchard, 1845 consists of derived cyclostome wasps, with 2000+ described valid species. Members of Opiinae are koinobiont endoparasitoids of dipterous larvae, some of which are agricultural pests, such as leaf-mining and fruit-infesting species. Therefore, opiine parasitoids are potentially valuable for biological control (Wharton 1997; Ovruski et al. 2000; Delrio et al. 2005; Wahyuni et al. 2017). The actual number of genera of Opiinae is 40+, but the boundaries of the genera *Opius* Wesmael, 1835 and *Eurytenes* Foerster, 1863 are not settled (e.g., Wharton 1987, 1988, 1997; Wharton and Norrbom 2013; van Achterberg 2023). We treat the genus *Sternaulopius* Fischer, 1965 as a valid genus by following Wharton (2006) and Sheng et al. (2019).

## ﻿Materials and methods

The specimens of *Areotetesconvergens* sp. nov. and *Neopiuscitrinus* were collected in a Malaise trap, while those of *Sternaulopiusmaculiferus* sp. nov., *S.macrophthalmos* and *Indiopiuschenae* were collected by using a net to sweep herbal vegetation. For identification of the subfamily Opiinae, see van Achterberg (1990, 1993, 1997); for references to the Opiinae, see Yu et al. (2016).

Morphological terminology follows van Achterberg (1988, 1993), including the abbreviations for wing venation. Measurements are taken as indicated by van Achterberg (1988): for the length and the width of a body part, the maximum length and width are taken, unless otherwise indicated. The length of the mesosoma is measured from the anterior border of the mesoscutum to the apex of the propodeum and of the first tergite from the posterior border of the adductor to the medio-posterior margin of the tergite.

Observations, photographic images, and descriptions were made either with a digital stereo microscope (VHX-1000, Keyence) and with a LEICA DMC2900 digital camera or with a LEICA M205 C microscope (Leica Geosystems AG). Images were stacked with Helicon Focus v. 7 software (Helicon Soft, Kharkiv, Ukraine). After stacking, illustrations were created using Adobe Photoshop CS5.1.

The type specimens are deposited in the Osaka Museum of Natural History (OMNH) in Osaka.

## ﻿Systematics

### ﻿Genus Areotetes

Taxon classificationAnimaliaHymenopteraBraconidae

van Achterberg & Li, 2013

7F350BBD-7179-5139-BAB3-A9A45C3FC693

Areotetes van Achterberg & Li, 2013: 39–51. Type species (by original designation): Areotetescarinuliferus Li & van Achterberg, 2013.

#### Diagnosis.

Basal carina of inner side of hind tibia long and slightly sinuate (Fig. 10); occipital carina present laterally; hypoclypeal depression distinct (Fig. 7); clypeus obtuse and truncate ventrally; malar sulcus absent; mandible normal and triangular; pronope absent and obsolescent (Fig. 8); precoxal sulcus finely crenulate or smooth (Fig. 4); medio-posterior depression of mesoscutum rather small or absent (Fig. 5); areola of propodeum distinct and with medio-longitudinal carina (Fig. 9); second submarginal cell of fore wing elongated (Fig. 2); vein m-cu of fore wing postfurcal; ovipositor sheath with very long setae (Fig. 6).

#### Distribution.

Palaearctic [China, Japan (new record), South Korea] and Oriental (China).

#### Biology.

Unknown.

##### ﻿Key to species of the genus *Areotetes* van Achterberg & Li

Notes: Modified after Li et al. (2013), with *Opiusnepalensis* Fischer, *Uteteslaevigatus* Weng & Chen and the species described in this paper added.

**Table:** 

1	Medio-posterior depression of mesoscutum absent; antenna of ♀ without white apical band; second metasomal tergite smooth; face dark brown or blackish	**2**
–	Medio-posterior depression of mesoscutum present, but small; antenna of ♀ with white apical or subapical band; second tergite distinctly costate-striate medially; face yellowish-brown or pale yellowish	**3**
2	Length of mesosoma 1.4–1.5× its height; propodeum without a medio-longitudinal carina posteriorly; hind femur 5.0–5.4× longer than its maximum width; vein 3-SR of fore wing 2.3–2.4× longer than vein 2-SR; [first metasomal tergite at least partly smooth and shiny]	***A.carinuliferus* Li & van Achterberg, 2013**
–	Length of mesosoma about 1.7× its height; propodeum with a medio-longitudinal carina posteriorly; hind femur about 4.0× longer than its maximum width; vein 3-SR of fore wing about twice longer than vein 2-SR	***A.laevigatus* (Weng & Chen, 2005)**
3	Vein 3-SR of fore wing about 1.7× longer than vein 2-SR and nearly straight; head dorsally (except stemmaticum and its surroundings) yellow; apical third of antenna of ♂ pale yellowish; pterostigma comparatively wide and short	***A.albiferus* Li & van Achterberg, 2013**
–	Vein 3-SR of fore wing 2.2–2.7× longer than vein 2-SR and weakly curved; head dorsally (except orbita) dark brown; apical third of antenna of ♂ dark brown; pterostigma comparatively narrow and longer	**4**
4	Vein r of fore wing emanating from basal 0.2 of pterostigma (Fig. 2); vein m-cu of fore wing distinctly converging towards vein 1-M posteriorly; [pedicel and third antennal segment yellowish; first metasomal tergite 1.6× longer than its apical width; length of hind femur 4.7× its maximum width]	***A.convergens* Han & van Achterberg, sp. nov.**
–	Vein r of fore wing emanating from basal 0.3 of pterostigma; vein m-cu of fore wing subparallel with vein 1-M posteriorly	**5**
5	Second metasomal tergite smooth (except for some indistinct striae medio-anteriorly); hind femur about 5.0× longer than its maximum width; vein 3-SR of fore wing about 2.2× longer than vein 2-SR; face more or less brownish; first tergite 1.6× longer than its apical width; apical antennal segments of ♀ ivory (but according to original description apical segments may be blackish); pedicel yellow and third antennal segment brown	***A.nepalensis* (Fischer, 1966), comb. nov.**
–	Second tergite finely to moderately striate or costate-striate medially; hind femur about 4.0× longer than its maximum width; vein 3-SR of fore wing about 2.5× longer than vein 2-SR; face largely yellowish; first tergite 1.4× longer than its apical width; apical antennal segments of ♀ brownish; pedicel and third antennal segment brown	***A.striatiferus* Li & van Achterberg, 2013**

### ﻿ Areotetes convergens

Taxon classificationAnimaliaHymenopteraBraconidae

Han & van Achterberg sp. nov.

4EE7935C-2F20-57D6-B0C3-F91285A46763

https://zoobank.org/CD30248E-A76B-41C0-878E-D42F3F1BE68A

Figs 1 , 2–13

#### Type material.

***Holotype***, ♀ (OMNH), “Japan (Ryuku): Oganeku, Yamato, Amamioshima Island, Kagoshima, 28.3593°N, 129.3441°E, 25.v.–15.vi.2019, MT [=Malaise trap], A. Yoshikawa & Shunpei Fujie leg., OMNH”.

#### Diagnosis.

**Figure 1.**
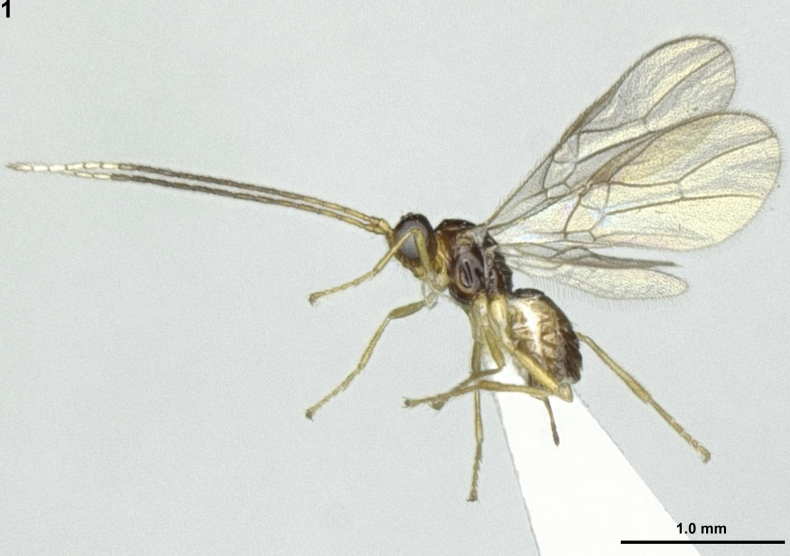
*Areotetesconvergens* Han & van Achterberg, sp. nov., holotype, ♀, Japan, habitus, lateral.

**Figures 2–13.**
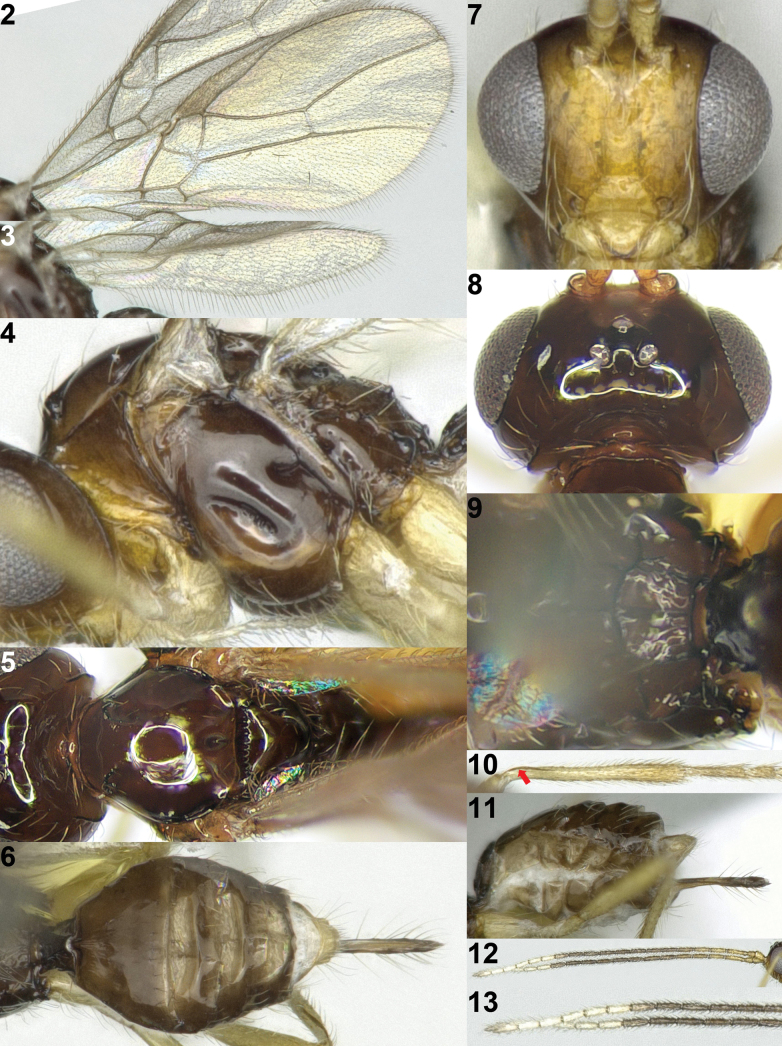
*Areotetesconvergens* Han & van Achterberg, sp. nov., holotype, ♀, Japan. **2** fore wing **3** hind wing **4** mesosoma lateral **5** mesosoma and head dorsal **6** metasoma dorsal **7** head anterior **8** head dorsal **9** propodeum dorsal **10** hind leg inner side **11** metasoma and ovipositor sheath lateral **12** antenna **13** apex of antenna. The arrow indicates carina on inner side of hind tibia.

Antenna of ♀ with white band (Figs 1, 12, 13; apical sixth segments); second metasomal tergite largely smooth except faintly striate-rugose medio-basally; clypeus 2.3× wider than its maximum height; pronope transverse elliptical; vein r of fore wing emanating from basal 0.2 of pterostigma (Fig. 2); vein m-cu of fore wing distinctly converging towards vein 1-M posteriorly; vein 3-SR of fore wing 2.4× longer than vein 2-SR and curved downward (Fig. 2); propodeum largely shiny and smooth but with a long medio-longitudinal carina and two diverging oblique carinae and area behind carinae with distinct areola (Fig. 9); setose part of ovipositor sheath about as long as first tergite (Fig. 11).

#### Description.

Holotype, female; length of body 1.6 mm, of fore wing 1.9 mm.

***Head*.** Antenna with 20 segments, 1.4× longer than body (Fig. 12); third segment 4.3× longer than its width and 1.3× longer than fourth segment, subapical segments 2.5× longer than its width; eye 2.9× longer than temple in dorsal view (Fig. 8); vertex, frons and occiput smooth and glabrous; face smooth and sparsely long setose (Fig. 7); clypeus 2.3× wider than its maximum height; clypeus shiny, smooth, remotely setose, and rather flat in lateral view, ventral margin of clypeus concave; hypoclypeal depression present; maxillary palp nearly 0.9× as long as height of head; malar sulcus absent; occipital carina interrupted dorsally (Fig. 8); mandible gradually widened basally, moderately setose and slightly twisted in lateral view without acute basal lamella (Figs 4, 7).

***Mesosoma*.** Mesosoma 1.4× longer than its height (Fig. 4); pronope distinct, elliptical (Fig. 5); pronotum smooth and sparsely setose along anterior and lateral margin; pronotal side and propleuron smooth and glabrous (Fig. 4), but smooth transverse carina present ventro-posteriorly; mesopleuron largely smooth and glabrous, but precoxal sulcus crenulate medially, medium-sized and oblique; epicnemial area smooth; mesopleural sulcus smooth; mesosternum smooth and moderately setose; anterior groove of metapleuron smooth, remaining area shiny, smooth and densely setose dorso-anteriorly and ventrally; notauli absent on mesoscutal disc, but a pair of crenulate impressions present anteriorly (Fig. 5); medio-posterior depression of mesoscutum round and shallow; mesoscutum shiny, smooth and sparsely setose; scutellar sulcus narrow and densely crenulate; scutellum smooth and rather flat in lateral view; propodeum smooth, but with a long medio-longitudinal carina and two diverging oblique two transverse carinae and area behind carinae with areola distinct (Fig. 9).

***Wings*.** Fore wing (Fig. 2): Pterostigma narrow elongate triangular, and gradually narrowed apically; vein r very short, angled with vein 3-SR and emanating from basal; veins 1-M and 1-SR+M straight; vein 3-SR curved downward, vein 3-SR 2.7× longer than vein 2-SR and subparallel with vein 2-M apically; r:3-SR:SR1 = 5:71:109; vein SR1 curved upwards; vein m-cu distinctly postfurcal and distinctly converging to vein 1-M posteriorly; second submarginal cell elongated (Fig. 2); first subdiscal cell closed; vein CU1b present. Hind wing (Fig. 3): narrow; vein 1r-m 0.4× longer than vein 1-M; veins m-cu and 2-M absent.

***Legs*.** Length of hind femur 4.7× its maximum width (Fig. 1); basal carina of inner side of hind tibia long and slightly sinuate (Figs 6, 10).

***Metasoma*.** First metasomal tergite 1.6× longer than its apical width, its surface rugose and slightly convex medio-basally in lateral view (Figs 1, 11); dorsope absent; second metasomal suture obsolescent (Fig. 6); second tergite shiny, smooth but faintly striae-rugose anteriorly and with shallow pair of depressions medio-basally; following tergites shiny, smooth and posteriorly setose; setose part of ovipositor sheath 1.6× longer than first tergite and with very long setae (Fig. 11).

***Colour*.** Body generally dark brown (Fig. 1); 6 apical segments of antenna, white; face, clypeus, mandible and pronotal side antero-ventrally, yellowish-brown; scape and basal fifth of antenna and legs, light yellowish; palpi, pale yellowish; pterostigma and vein of wings, light greyish-brown; wings, hyaline.

#### Distribution.

Japan (Ryuku Islands).

#### Biology.

Unknown.

#### Etymology.

From “con-” (Latin for together) and “vergo” (Latin for incline or turn toward) because of the posteriorly converging veins 1-M and m-cu of the fore wing.

#### Remarks.

The new species belongs to the genus *Areotetes* van Achterberg & Li because it has a distinct carina on the inner side of the hind tibia, clypeus slightly concave ventrally with a thick ventral margin, and the propodeum with a long medio-longitudinal carina and a distinct areola. It does not run in the keys by Li et al. (2013) and Tobias (1998). In Fischer (1972, 1987) it runs to the subgenus Utetes Foerster and to *O.kamikochiensis* Fischer, 1963 from Japan and *O.sanguanus* Fischer, 1966 from Nepal, respectively.

*Opiuskamikochiensis* Fischer differs from the new species by having the first metasomal tergite about as long as wide apically (1.6× in *A.convergens*), vein 1-SR of fore wing comparatively long (short), antenna with 25 segments (20 segments), vein r of fore wing longer and emanating from near basal third of pterostigma (very short and from basal 0.2 of pterostigma) and pterostigma wider (narrower). *Opiussanguanus* Fischer differs from the new species as follows: vein m-cu of fore wing comparatively far postfurcal (narrowly postfurcal in *A.convergens*), pterostigma wider (narrower), vein r emanating from basal 0.3 of pterostigma (from 0.2 of pterostigma) and third antennal segment brown (yellow). Actually, it is much more similar to *O.nepalensis* Fischer, 1966 from Nepal to which it does not run in Fischer (1987) because of the weird choice of not considering variation between mesosoma 1.4 or 1.5× longer than height in lateral view; the few differences between both species are summarized in the key.

### ﻿Genus Indiopius

Taxon classificationAnimaliaHymenopteraBraconidae

Fischer, 1966

61264097-9C3F-59EB-9CCF-F742B4853388

Indiopius Fischer, 1966: 154–155. Type species (by original designation): Indiopiushumillimus Fischer, 1966.

#### Diagnosis.

Marginal cell of fore wing open apically (Fig. 15); first subdiscal cell of fore wing open (Fig. 15); veins m-cu and r-m of fore wing absent (Fig. 15); vein cu-a of hind wing absent (Fig. 15); clypeus transverse (Fig. 18); occipital carina entirely absent (Figs 20, 21); first to third metasomal tergites more or less coriaceous or rugulose.

#### Distribution.

Palaearctic including West Palaearctic, East Palaearctic [Japan (new record)] and Oriental.

#### Biology.

Unknown.

##### ﻿Key to species of the genus *Indiopius* Fischer

**Table:** 

1	Frons with elongate depression between stemmaticum and eyes; vein 2-1A of fore wing not pigmented; vein cu-a of fore wing postfurcal by more than its width; vein 1-R1 about as long as distance between apex of vein 1-R1 and apex of fore wing; India	***I.fischeri* Samiuddin & Ahmad, 2009**
–	Frons without elongate depression between stemmaticum and eyes, at most with punctures; vein 2-1A of fore wing more or less pigmented; vein cu-a of fore wing postfurcal by its width or interstitial; vein 1-R1 of fore wing 1.2–8.0× longer than distance between apex of vein 1-R1 and apex of fore wing, but about equal in *I.humillimus* and *I.yilmazae*	**2**
2	Vein 1-R1 of fore wing about 1.8× longer than pterostigma and vein 1-R1 of fore wing about 8× longer than distance between its apex and apex of fore wing; posterior margin of pterostigma slightly curved; vein 1-SR absent; Turkmenistan	***I.turcmenicus* Tobias, 1986**
–	Vein 1-R1 of fore wing 1.0–1.3× longer than pterostigma and vein 1-R1 of fore wing 1.0–4.0× longer than distance between its apex and apex of fore wing; posterior margin of pterostigma straight; vein 1-SR present, but sometimes narrowly so	**3**
3	First metasomal tergite about 1.2× longer than wide apically; between stemmaticum and eyes with a setiferous puncture; antenna of ♀ with about 17 segments; [body brown, but head and mesosoma (except propodeum) and apex of metasoma dark brown]; Vietnam	***I.saigonensis* Fischer, 1966**
–	First tergite about as long as wide apically; between stemmaticum and eyes without a setiferous puncture; antenna of ♀ with 18–19 segments	**4**
4	Vein 1-R1 of fore wing about as long as distance between its apex and apex of fore wing	**5**
–	Vein 1-R1 of fore wing 1.5–3.0× longer than distance between its apex and apex of fore wing	**6**
5	Mesoscutum and head dorsally black; metasoma reddish-brown, but its apex black; antennal segments of ♀ 18; Turkey	***I.yilmazae* Fischer & Beyarslan, 2011**
–	Mesoscutum and head dorsally yellow; metasoma yellow but its apex dark brown; antennal segments of ♀ 19; India	***I.humillimus* Fischer, 1966**
6	Width of scutellar sulcus 0.3 times length of scutellum (Fig. 17); fore femur wider than middle femur; [precoxal sulcus distinctly crenulate; face medially paler than dorso-laterally; vein 1-R1 of fore wing about 1.5× longer than distance between its apex and apex of fore wing]; China (Hunan)	***I.chenae* van Achterberg & Li, 2013**
–	Width of scutellar sulcus 0.1–0.2 times length of scutellum; fore femur about as wide as middle femur	**7**
7	Vein 1-R1 of fore wing about 1.5× longer than distance between its apex and apex of fore wing; vein 3-SR+SR1 less curved and pointing behind apex of vein 1-R1; notauli indistinctly impressed anteriorly; precoxal sulcus smooth or finely crenulate; [antennal segments of ♀ 18; of ♂ 20]; Mediterranean, Cape Verde Islands	***I.cretensis* Fischer, 1983**
–	Vein 1-R1 of fore wing about 3× longer than distance between its apex and apex of fore wing; vein 3-SR+SR1 more curved and pointing towards apex of vein 1-R1; notauli distinctly impressed anteriorly; precoxal sulcus distinctly crenulate; [antennal segments of ♀ 18–19]; China (Fujian)	***I.alutacius* Weng & Chen, 2001**

### ﻿ Indiopius chenae

Taxon classificationAnimaliaHymenopteraBraconidae

van Achterberg & Li, 2013

4400F237-7EA5-5617-BED4-285FD83D03A5

Figs 14 , 15–25

Indiopius chenae van Achterberg & Li, 2013: 66–69.

#### Material.

1 ♂ (OMNH), “Japan (Honshu): Oyamada-chou, Kawachinagano, Osaka, 34.4509°N, 135.5504°E, 23.ix.2017, SW[=collected by sweeping], Shumei Fujie leg., OMNH”; 1 ♀ (OMNH), “Japan (Honshu): Yamazakichou Koudani, Shisou, Hyogo, 35.0238°N, 134.5619°E, 16.ix.2019, SW[=collected by sweeping], Shumei Fujie, OMNH”; 1 ♀ (OMNH), “Japan (Honshu): Oyamada-chou, Kawachinagano, Osaka, 34.4509°N, 135.5504°E, 5.ix.2016, SW[=collected by sweeping], Takao Aoki leg., OMNH”.

#### Diagnosis.

Antenna with 19 segments (Fig. 24); between stemmaticum and eyes sparsely setose without depression; occipital carina entirely absent (Figs 19–21); clypeus 4.3× longer than its maximum height (Fig. 19); mandible gradually widened basally; vein 1-R1 of fore wing 1.7× longer than distance between apex of vein 1-R1 and apex of fore wing; vein cu-a of fore wing interstitial (Fig. 15); maxillary palpi 0.5× as long as height of head; scutellar sulcus robust and distinctly crenulate medially (Fig. 17); fore femur somewhat wider than middle femur; strong dorsal carinae separated up to posterior of first metasomal tergite; first metasomal tergite entirely granulate-rugose (Figs 18, 23).

#### Re-description.

**Female**; length of body 1.6 mm, of fore wing 1.3–1.6 mm, male; length of body 1.3 mm, of fore wing 1.5 mm.

***Head*.** Antenna with 19 segments, 1.1× longer than body (Fig. 24); third segment 3.1× longer than its width and 1.2× longer than fourth segment (Figs 24, 25); middle flagellar segment 2.9× longer than its width; depression of frons slightly present near antennal sockets; eye as long as temple (Fig. 20); vertex, frons, stemmaticum and occiput smooth and glabrous; face shiny, smooth and moderately setose (Fig. 19); median keel of frons slightly present; clypeus 4.3× wider than its maximum height; clypeus narrow, trapezoid-shaped, shiny, smooth, moderately setose and flat in lateral view, and its ventral margin straight; hypoclypeal depression slightly present; maxillary palpi 0.5× as long as height of head; malar sulcus present and short; occipital carina absent (Figs 20, 21); mandible slightly twisted, moderately setose and slightly widened basally.

**Figure 14.**
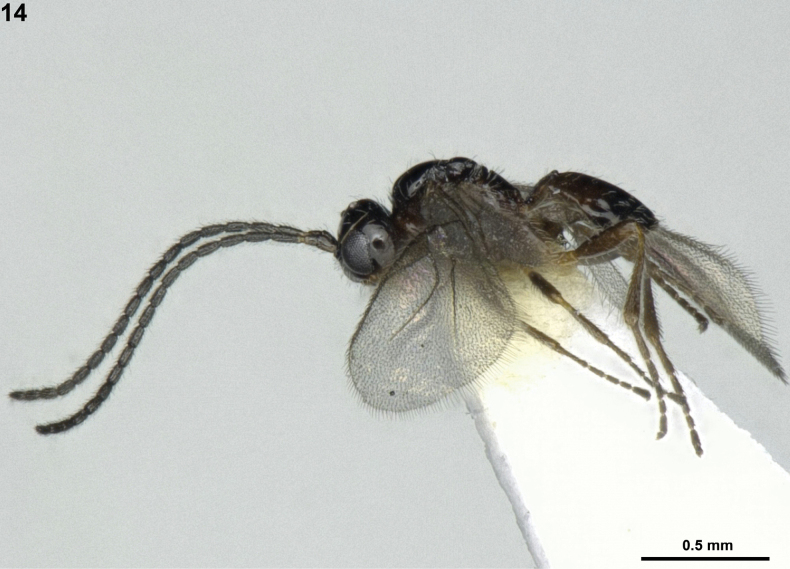
*Indiopiuschenae* van Achterberg & Li, ♂, Japan, habitus, lateral.

***Mesosoma*.** Mesosoma 1.3× longer than its height (Fig. 16); pronope absent; pronotal side smooth, glabrous, and anterior and posterior groove of pronotal side smooth (Fig. 16); propleuron shiny, smooth and rather moderately setose; mesopleuron largely smooth, but precoxal sulcus deeply narrowly crenulate; epicnemial area narrowly crenulate ventrally, remaining area smooth; mesopleural sulcus smooth; mesosternum rather sparsely setose; anterior groove of metapleuron smooth; metapleuron largely shiny, smooth and densely setose posteriorly; notauli absent on disc of mesoscutum, but deeply crenulate anteriorly (Fig. 17); medio-posterior depression of mesoscutum absent; mesoscutum smooth and glabrous; scutellar sulcus straight, relatively short, medium-sized and densely crenulate medially, but reduced laterally; scutellum smooth, glabrous and not protruding above level of mesoscutum in lateral view (Figs 16, 17); propodeum smooth and glabrous without any carinae (Figs 18, 22).

**Figures 15–25.**
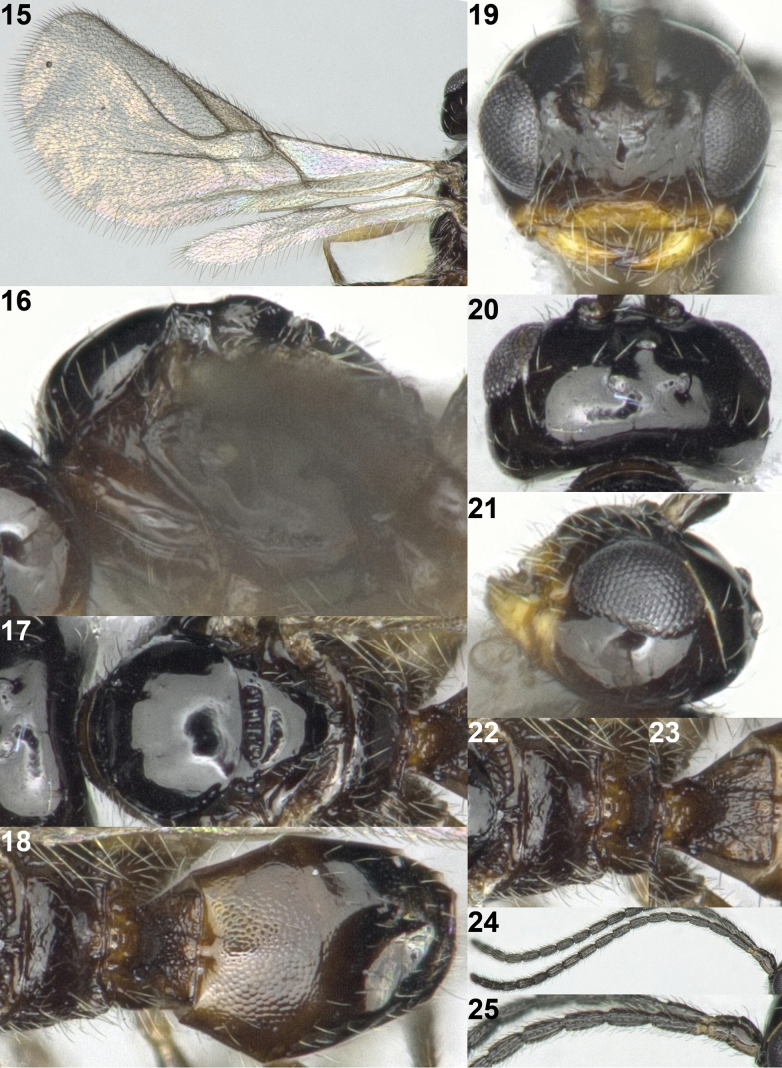
*Indiopiuschenae* van Achterberg & Li, ♂, Japan. **15** wings **16** mesosoma lateral **17** mesosoma dorsal **18** propodeum and metasoma dorsal **19** head anterior **20** head dorsal **21** head lateral **22** propodeum dorsal **23** first metasomal tergite dorsal **24** antenna **25** base of antenna.

***Wings*.** Fore wing (Fig. 15): Pterostigma triangular; vein 1-M slightly curved; vein 1-SR+M curved downwards; vein 3-SR+SR1 strongly curved and pointing towards apex of vein 1-R1; vein 1-R1 of fore wing about 1.7× longer than distance between its apex and apex of fore wing; vein 3-SR converging with vein 2-M; vein 1-SR short; vein cu-a interstitial; first subdiscal cell open. Hind wing: entirely narrow and subparallel-sided; vein cu-a absent; vein m-cu absent; vein 2-M pigmented basally and reduced apically.

***Legs*.** Length of hind femur 3.9× its maximum width; fore femur slightly wider than middle femur, nearly 1.1× wider than maximum width of middle femur.

***Metasoma*.** First metasomal tergite as long as its apical width, its surface reticulate-rugose with strong dorsal carinae, and slightly convex medially in lateral view (Fig. 14); dorsope absent; second metasomal suture obsolescent dorsally (Fig. 18); second tergite granulate and glabrous, with pair of triangular depressions basally; third tergite granulate antero-medially and remaining area smooth; following tergites shiny, smooth, and moderately setose posteriorly.

***Colour*.** Body generally dark brown to black (Fig. 14); clypeus and mandible, light brown; pronotal side ventrally and first to third anteriorly metasomal tergites, brown; antenna and legs, dark brown (except legs darker brown dorsally); pterostigma and veins of wings, greyish-brown; wings, hyaline.

#### Distribution.

Japan (Honshu; new record), China (Fujian).

#### Biology.

Unknown.

### ﻿Genus Neopius

Taxon classificationAnimaliaHymenopteraBraconidae

Gahan, 1917

21B334D2-24B2-51C7-B0AB-64E1102E0F37

Neopius Gahan, 1917: 203. Type species (by original designation): Neopiuscarinaticeps Gahan, 1917 (= Opiusrudis Wesmael, 1835). Synonymized by Quicke et al. (1997) with Opius Wesmael, 1835 and restored as valid genus by Li et al. (2013).

#### Diagnosis.

Occipital carina completely crenulate in dorsal and lateral view (Figs 32, 33); frons distinctly granulate (Fig. 32); mandible normal and symmetrically widened basally (Fig. 33); hypoclypeal depression distinct (Fig. 31); precoxal sulcus sculptured (Fig. 28); pronotum sculptured; notauli sculptured at least half of mesoscutum (Fig. 29); medio-posterior depression of mesoscutum present; mesoscutum and mesopleuron largely granulate; ovipositor sheath short.

#### Distribution.

Holarctic, including Japan (new record) and South Korea.

#### Biology.

Endoparasitoids of Agromyzidae larvae (including species *Agromyzamegalopsis* Hering, 1933 and *Agromyzanigripes* Meigen, 1830).

##### ﻿Key to species of the genus *Neopius* Gahan

Notes: Modified after Sheng et al. (2019).

**Table:** 

1	Face yellowish-brown (Fig. 31); notauli gradually reduced posteriorly (Fig. 29); vein m-cu of fore wing shorter than vein 2-CU1 (Fig. 27); occiput comparatively straight in dorsal view (Fig. 32)	***N.citrinus* Sheng & Chen, 2019**
—	Face largely dark brown; notauli complete, reaching medio-posterior depression of mesoscutum; vein m-cu of fore wing about 1.5× longer than vein 2-CU1; occiput concave in dorsal view	***N.rudis* (Wesmael, 1835)**

### ﻿ Neopius citrinus

Taxon classificationAnimaliaHymenopteraBraconidae

Sheng & Chen, 2019

64196567-807F-5E9A-B875-3030BDBC9DE8

Figs 26 , 27–36

Neopius citrinus Sheng & Chen, 2019: 592–595.

#### Material.

1 ♀ (OMNH), “Japan (Honshu): Nakaikemi Wetlands, Kashimagari, Tsuruga, Fukui, 35.6594°N, 136.0884°E, 24.v.–17.vi.2016, MT [=Malaise trap], Asato Noishiki leg., OMNH”.

#### Diagnosis.

Face yellow (Fig. 31); notauli nearly complete, gradually reduced posteriorly; occiput comparatively straight in dorsal view (Fig. 32); head largely granulate; scutellar sulcus comparatively robust (Fig. 29); propodeum reticulate-rugose with a medio-longitudinal carina; second and third tergites granulate.

#### Re-description.

**Female**; length of body 2.8 mm, of fore wing 3.0 mm.

***Head*.** Antenna with 29 segments (Fig. 25; broken and lost); third segment 2.3× longer than its width, 1.2× longer than fourth segment; eye 1.5× longer than temple in dorsal view (Fig. 23); vertex granulate and sparsely setose; frons and occiput shiny and granulate; face densely punctate and setose (Fig. 21); clypeus 2.5× wider than its maximum height; clypeus densely setose, its ventral margin slightly protruding downward; hypoclypeal depression distinct; maxillary palpi nearly 0.7× as long as height of head; malar sulcus absent; occipital carina completely present and crenulate, (Figs 29, 32); mandible gradually widened basally, moderately setose and hardly twisted in lateral view without acute basal lamella (Fig. 33).

**Figure 26.**
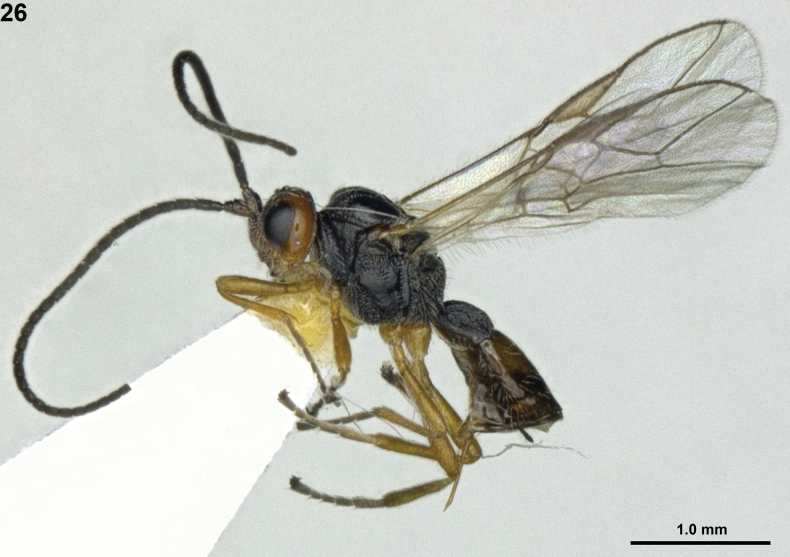
*Neopiuscitrinus* Sheng & Chen, ♀, Japan, habitus, lateral.

**Figures 27–36.**
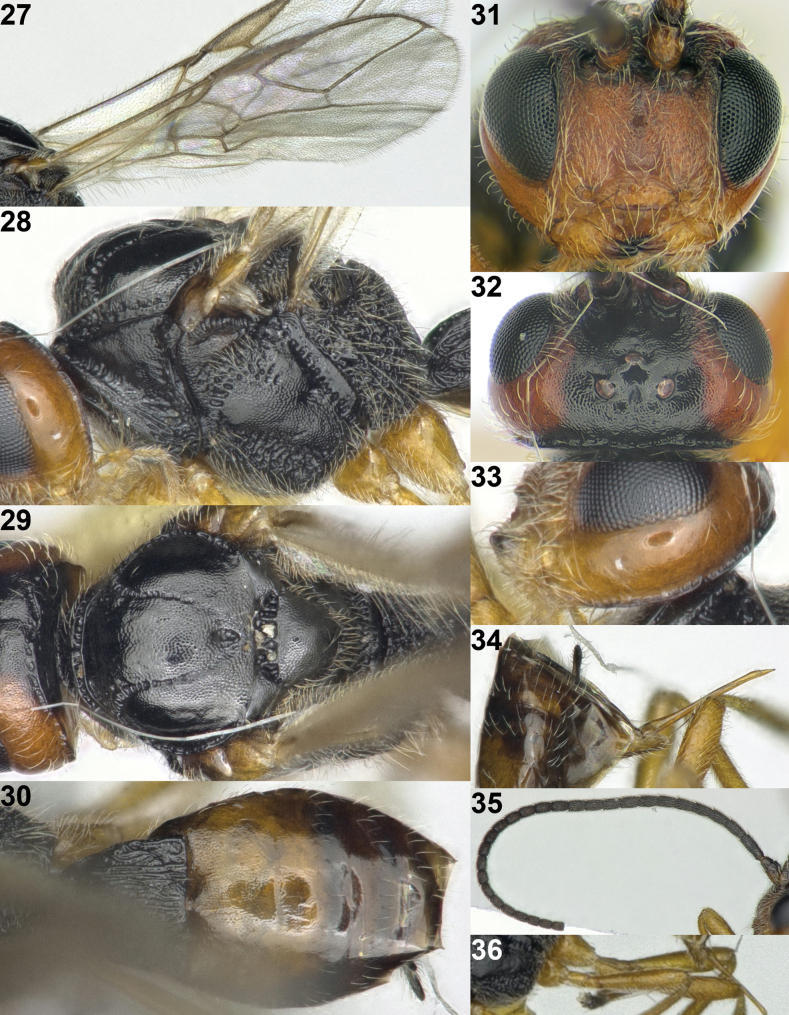
*Neopiuscitrinus* Sheng & Chen, ♀, Japan. **27** fore wing **28** mesosoma lateral **29** mesosoma dorsal **30** metasoma dorsal **31** head anterior **32** head dorsal **33** head lateral **34** ovipositor lateral **35** antenna **36** hind femur.

***Mesosoma*.** Mesosoma 1.3× longer than its height (Fig. 29); pronope absent but transverse crenulated groove and sparsely setose along lateral margin (Fig. 30); pronotal side granulate with ventral crenulated groove; propleuron granulate (Fig. 29); mesopleuron largely coriaceous, but precoxal sulcus crenulate, wide, oblique and reaching anterior area (converging epicnemial area and dorsal crenulate carina); epicnemial area crenulate; mesopleural sulcus crenulate; mesosternum densely setose; anterior groove of metapleuron crenulate, remaining area reticulate-rugose, shiny and densely setose; notauli distinctly crenulate antero-medially, but gradually obsolescent posteriorly (Fig. 29); medio-posterior depression of mesoscutum large, elliptical and deep; mesoscutum coriaceous with few setae along the notaulic course; scutellar sulcus medium-sized, moderately crenulate and gradually narrowed laterally; scutellum granulate and rather flat in lateral view; propodeum entirely reticulate-rugose and densely setose with a short medio-longitudinal carina.

***Wings*.** Fore wing (Fig. 27): Pterostigma triangular and gradually narrowed apically; vein r sublinear with vein 3-SR; vein 1-M nearly straight; vein 1-SR+M straight; vein 2-SR slightly sinuate and oblique; vein 3-SR 1.5× longer than vein 2-SR; r:3-SR:SR1 = 5:18:37; vein SR1 straight; vein m-cu distinctly postfurcal; first subdiscal cell open; vein CU1b short and incomplete. Hind wing (Fig. 28): vein m-cu absent; vein 2-M absent.

***Legs*.** Length of hind femur 4.4× its maximum width (Fig. 36).

***Metasoma*.** First metasomal tergite 0.9× as long as its apical width, its surface striate-rugose and slightly convex medio-basally in lateral view (Figs 26, 30); dorsope absent; second metasomal suture obsolescent (Fig. 30); second tergite shiny and granulate, with shallow pair of depressions medio-basally; third tergite granulate medially and following tergites shiny and smooth, with band of setae posteriorly; setose part of ovipositor sheath 0.5× longer than first tergite (Fig. 34).

***Colour*.** Body generally black (Fig. 26); head yellowish-brown but frons and vertex black medially (Fig. 32); legs and ovipositor, light brown; second and half of basal third tergites, brown; pterostigma and vein of wings, light brown; wings, subhyaline.

#### Distribution.

Japan (Honshu; new record), China (Heilongjiang, Jilin and Liaoning).

#### Biology.

Unknown.

### ﻿Genus Sternaulopius

Taxon classificationAnimaliaHymenopteraBraconidae

Fischer, 1965

48CB1F0D-86EF-54E0-AE98-F9EB8C14C2B7

Sternaulopius Fischer, 1965: 311; Wharton, 2006: 317. Type species (monobasic and by original designation). Sternaulopiusbisternaulicus Fischer, 1965.

#### Diagnosis.

Below precoxal sulcus with a distinct and sculptured second sulcus (= sternaulus; Figs 39, 50); malar space largely smooth, in East Asian species deeply impressed (Figs 43, 54, 56); mandible gradually widened basally and without basal lamella; occipital carina absent medio-dorsally; medio-posterior depression of mesoscutum present as small point-like depression (Afrotropical spp.) or part of notauli and medium-sized (Asian spp.; Fig. 40); propodeum coarsely reticulate-rugose (Figs 45, 51); dorsope of first tergite deep (van Achterberg 1993, pl. 36) or shallow (Asian sp.; Fig. 41); setose part of ovipositor sheath 0.3–1.0× as long as first tergite.

#### Distribution.

Palaearctic [Japan (new record); China (Jilin)], Oriental [China (Sichuan)] and Afrotropical (Burundi, Cameroon, Democratic Republic of Congo, Kenya, Madagascar) regions. The European records concern *Biophthora* Foerster, 1863 and *Sternaulopius* s. str. has not yet been found in Europe.

#### Biology.

Parasitoids of fruit-infesting dipterous larvae of Tephritidae (*Ceratitis* MacLeay, 1829 and *Trirhithrum* Bezzi, 1918).

##### ﻿Key to species of the genus *Sternaulopius* Fischer

**Table:** 

1	Hypoclypeal depression absent; mesoscutum densely setose; precoxal sulcus and sternaulus gradually converging posteriorly; Afrotropical (Madagascar)	***S.duplicatus* Wharton, 2006**
–	Hypoclypeal depression present (Fig. 43); mesoscutum densely or sparsely setose; precoxal sulcus subparallel to precoxal sulcus posteriorly (Fig. 39; van Achterberg 1993, pl. 36)	**2**
2	Posterior half of notauli smooth and shallowly impressed or absent; medio-posterior depression of mesoscutum small, point-like, far removed from notauli (van Achterberg 1993, pl. 36); occipital carina entirely smooth and narrow laterally; dorsope of first tergite deep; malar space without wide depression; distal half of pterostigma slender (van Achterberg 1993, pl. 36); Afrotropical (continental Africa)	***S.bisternaulicus* Fischer, 1965**
–	Posterior half of notauli crenulate and deep (Fig. 40); medio-posterior depression of mesoscutum medium-sized and more or less part of notauli (Fig. 40); occipital carina partly crenulate and wide laterally (Fig. 39); dorsope of first tergite shallow (Fig. 41); malar space with wide depression (Fig. 43); distal half of pterostigma robust (Fig. 38); East Palaearctic	**3**
3	Vein 3-SR of fore wing 1.3–1.4× vein 2-SR; area below pterostigma subhyaline; vein 1-M of fore wing curved; vein m-cu of fore wing postfurcal; length of eye in dorsal view 2.2–2.6× temple	***S.macrophthalmos* Sheng & Chen, 2019**
–	Vein 3-SR of fore wing as long as vein 2-SR (Fig. 38) area below pterostigma with brownish patch (Fig. 38); vein 1-M of fore wing straight or nearly so (Fig. 38); vein m-cu of fore wing antefurcal; length of eye in dorsal view 1.9× temple (Fig. 44)	***S.maculiferus* Han & van Achterberg, sp. nov.**

### ﻿ Sternaulopius maculiferus

Taxon classificationAnimaliaHymenopteraBraconidae

Han & van Achterberg sp. nov.

A5F8C0AB-8811-56F8-9BEC-212F42FA5F97

https://zoobank.org/D7A31E30-4F94-4B00-86CF-C18117F0D72D

Figs 37 , 38–47

#### Type material.

***Holotype***, ♀ (OMNH), “Japan (Honshu): Nochino, Ono, Fukui, 35.9492°N, 136.6868°E, 5.viii.2011, SW[=collected by sweeping], Shunpei Fujie leg., OMNH”.

#### Diagnosis.

Vein 3-SR of fore wing as long as vein 2-SR (Fig. 38); below pterostigma with brownish patch; first subdiscal cell subparallel-sided (Fig. 38); hypoclypeal depression distinct (Fig. 43); eye 1.9× longer than temple in dorsal view (Fig. 44); mesoscutum densely setose (Fig. 40); notauli complete and crenulate; medio-posterior depression of mesoscutum medium-sized and part of notauli (Fig. 40); precoxal sulcus oblique and wide crenulate (Fig. 39); sternaulus crenulate and subparallel-sided with precoxal sulcus; propodeum with short medio-longitudinal carina and transverse carinae, and area behind carinae coarsely reticulate (Fig. 45).

**Figure 37.**
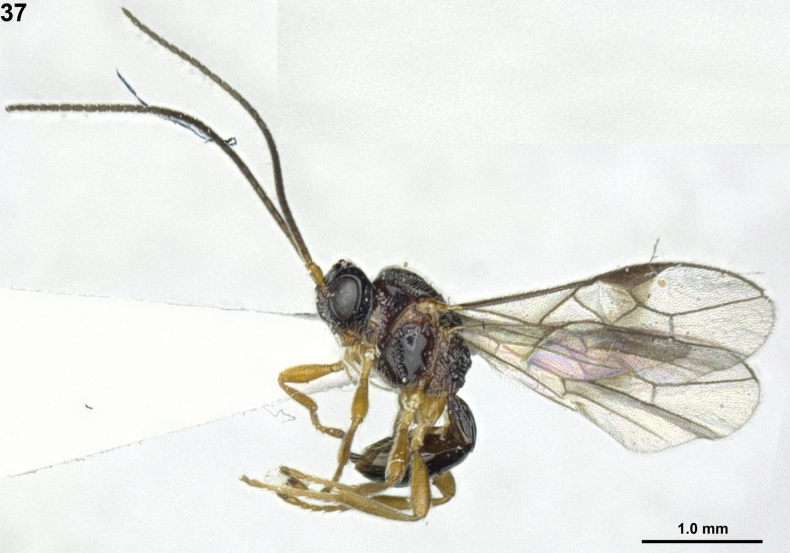
*Sternaulopiusmaculiferus* Han & van Achterberg, sp. nov., holotype, ♀, Japan, habitus, lateral.

#### Description.

Holotype, female; length of body 3 mm, of fore wing 2.8 mm.

***Head*.** Antenna with 31 segments and as long as body (Fig. 47); third segment 2× longer than wide, 1.3× longer than fourth segment; eye 1.9× longer than temple in dorsal view (Fig. 44); vertex, frons and occiput smooth and glabrous; face densely coarsely punctate and densely setose (Fig. 43); median keel present on face, smooth (Fig. 43); clypeus 2× wider than its maximum height; clypeus faintly punctate, sparsely setose, and protruding in lateral view; hypoclypeal depression present; length of maxillary palpi nearly 0.9× as long as height of head; malar sulcus present; occipital carina interrupted dorsally (Fig. 44); mandible gradually widened basally and densely setose without acute basal lamella.

***Mesosoma*.** Mesosoma 1.4× longer than its height (Fig. 39); pronope absent (Fig. 40); pronotal side with crenulate carina anteriorly and posteriorly (Fig. 39); propleuron rugose and densely setose without oblique carina; mesopleuron largely smooth, but precoxal sulcus crenulate and wide, oblique, reaching anterior part (wide and crenulate area in epicnemial area); sternaulus crenulate and subparallel with precoxal sulcus (Fig. 39); mesopleural sulcus widely crenulate; mesosternum densely setose; anterior groove of metapleuron crenulate and rather densely setose ventrally, remaining area rugose; notauli complete, and crenulate on disc of mesoscutum and reaching mesoscutum posteriorly (Fig. 40); medio-posterior depression of mesoscutum present and part of notauli; mesoscutum rather densely, weakly punctate and densely setose; scutellar sulcus wide, crenulate and curved; scutellum smooth setose and slightly convex in lateral view, but not protruding above level of mesoscutum; propodeum glabrous and reticulate-rugose with short medio-longitudinal carina and diverging oblique two transverse carinae, and area behind carinae coarsely reticulate (Fig. 45).

**Figures 38–47.**
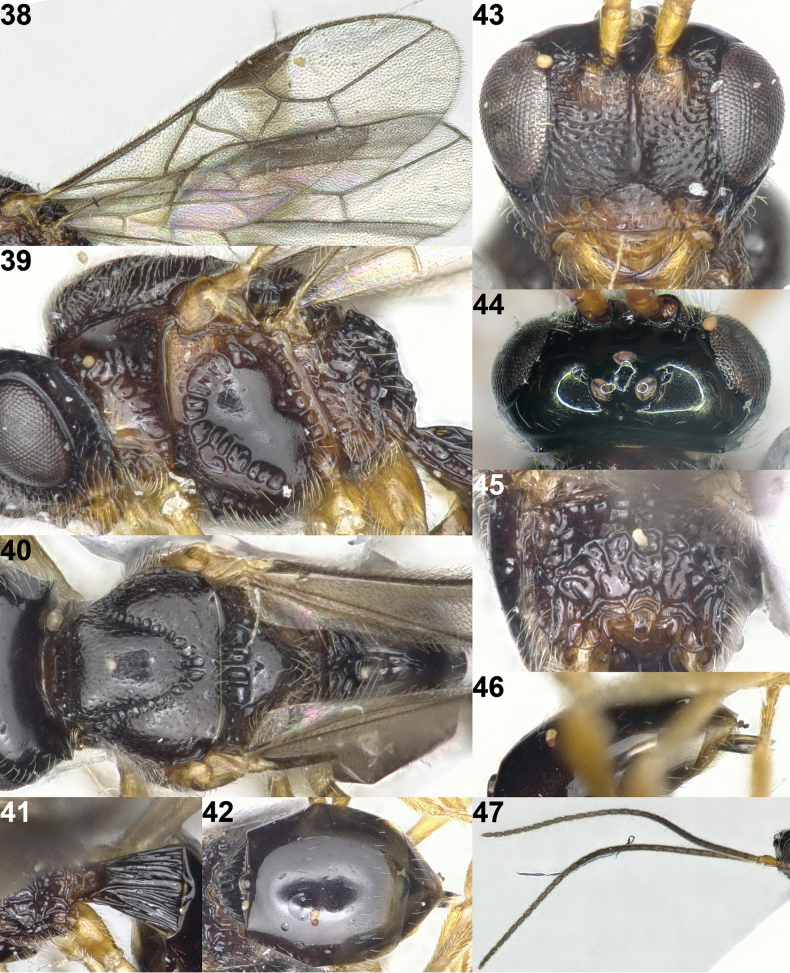
*Sternaulopiusmaculiferus* Han & van Achterberg, sp. nov., holotype, ♀, Japan. **38** wings **39** mesosoma lateral **40** mesosoma dorsal **41** first metasomal tergite dorsal **42** metasoma dorsal **43** head frontal **44** head dorsal **45** propodeum dorsal **46** ovipositor lateral **47** antenna.

***Wings*.** Fore wing (Fig. 38): Pterostigma wide, triangular, and slightly convex anteriorly; vein r gradually merging in vein 3-SR; vein 1-M straight; vein 1-SR+M sinuate; vein 2-SR almost straight, as long as vein 3-SR (1.1× longer than vein 3-SR); r:3-SR:SR1 = 5:11:30; vein SR1 straight; vein m-cu distinctly antefurcal and converging to vein 1-M posteriorly; second submarginal cell short (Fig. 38); first subdiscal cell closed and subparallel-sided; vein CU1b present; vein CU1a almost completely pigmented. Hind wing: vein 1r-m 0.8× as long as vein 1-M; vein m-cu pigmented and curved basally; vein 2-M pigmented.

***Legs*.** Length of hind femur 3.1× its maximum width; fore and hind femora robust (Fig. 37).

***Metasoma*.** First metasomal tergite as long as its apical width, its surface densely striate-rugose and in lateral view convex medially (Fig. 41); shallow dorsope present (Figs 39, 41); second metasomal suture absent (Fig. 42); second tergite shiny and smooth, but with pair of shallow depressions medio-basally; following tergites shiny and smooth, with row of setae posteriorly; setose part of ovipositor sheath 0.3× as long as first tergite (Fig. 46).

***Colour*.** Body generally black (Fig. 37); ventral margin of clypeus, dorsal part of epicnemial area, ventral part of pronotal side and anterior part of metapleuron, brown; scape and pedicel of antenna, mandible (except tips of mandible), tegulae and legs, brownish-yellow; palpi, pale yellowish; pterostigma, veins and spot below pterostigma, more or less dark brown; wings, subhyaline.

#### Distribution.

Japan (Honshu).

#### Biology.

Unknown.

#### Etymology.

From “macula” (Lain for patch) and “ferus” (Latin for carrying) because of the brownish patch below the pterostigma.

#### Remarks.

This new species runs to the genus *Sternaulopius* Fischer because of the distinct sternaulus below the precoxal sulcus, the shallow dorsope on the first metasomal tergite, the coarsely rugose propodeum with distinct carinae, and the normal mandible (i.e., without basal lamella or tooth). However, it does not run in the key to *Opius* s.l. by Tobias (1998) by having the medio-posterior depression of mesoscutum connected to the notauli, the distinct hypoclypeal depression, the broadly sculptured precoxal sulcus and the area behind the carinae on the propodeum reticulate and with a short medio-longitudinal carina anteriorly. In the key by Sheng et al. (2019) to *Sternaulopius*, it does not run well either by having the distinct hypoclypeal depression, length of eye 1.9× temple in dorsal view, the densely setose mesoscutum, the distinctly sinuate vein 1-SR+M of the fore wing, and vein 2-SR of fore wing 1.1× longer than vein 3-SR.

### ﻿ Sternaulopius macrophthalmos

Taxon classificationAnimaliaHymenopteraBraconidae

Sheng & Chen, 2019

89C8D766-321B-5C33-975E-B7B7C48310F2

Figs 48 , 49–57

Sternaulopius macrophthalmos Sheng & Chen, 2019: 595–598.

#### Material.

1 ♂ (OMNH), “Japan (Honshu): Oyamada-chou, Kawachinagano, Osaka, 34.4509°N, 135.5504°E, 4.xii.2018, SW [=collected by sweeping], Shumei Fujie leg., OMNH”.

#### Diagnosis.

Antenna with 24 segments (Fig. 53); hypoclypeal depression distinct (Fig. 54); malar space depressed (Figs 50, 54, 56); dorsope slightly impressed (Figs 50, 51); precoxal sulcus and sternaulus distinctly crenulate, absent posteriorly and subparallel posteriorly (Fig. 50); medio-posterior depression rather large and round (Fig. 51); length of eye in dorsal view 2.2× temple (male; Fig. 55); mesoscutum shiny and densely setose (Fig. 51); vein 1- M of fore wing slightly curved (Fig. 49).

#### Re-description.

**Male**; length of body 2.1 mm, of fore wing 2.2 mm.

***Head*.** Antenna with 24 segments and 1.1× longer than body (Fig. 53); third segment 1.3× longer than fourth segment; eye 2.2× longer than temple (Fig. 55); vertex, frons and occiput smooth and glabrous; face faintly and moderately punctate and sparsely setose (Fig. 54); median keel present on face, smooth; clypeus 1.8× wider than its maximum height; clypeus faintly punctate, and its ventral margin pointed downward; hypoclypeal depression present; maxillary palp 0.7× longer than height of head; malar space with a wide depression; occipital carina interrupted dorsally (Figs 55, 56); mandible gradually widened basally.

***Mesosoma*.** Mesosoma 1.4× longer than its height (Fig. 50); pronope elliptical (Figs 51, 55); crenulate carina wide posteriorly on pronotal side (Fig. 50); propleuron smooth and moderately setose; mesopleuron largely smooth with setae dorsally and ventro-posteriorly, but precoxal sulcus crenulate and wide, oblique, reaching anterior part (wide and crenulate carina in epicnemial area); sternaulus crenulate and subparallel with precoxal sulcus (Fig. 50); epicnemial area crenulate; mesopleural sulcus wide and crenulate; mesosternum rather moderately setose; anterior groove of metapleuron crenulate and rather densely setose, remaining area rugose and setose; notauli narrowly crenulate on disc of mesoscutum and partly absent posteriorly, not reaching medio-posterior depression of mesoscutum (Fig. 51); medio-posterior depression of mesoscutum rather large, round and shallow; mesoscutum more or less densely, superficially punctate and densely setose; scutellar sulcus wide and crenulate; scutellum superficially punctate and slightly convex in lateral view, but not protruding above level of mesoscutum; propodeum rugose with indistinctly short medio-longitudinal carina, two diverging oblique transverse carinae behind medio-longitudinal carina, and remaining area reticulate-rugose (Fig. 51).

***Wings*.** Fore wing (Fig. 49): Pterostigma wide, wide elliptical; vein 1-M of fore wing slightly curved basally; vein 1-SR+M almost straight; vein r angled with vein 3-SR; vein 3-SR distinctly longer than vein 2-SR (1.3× longer than vein 2-SR); vein 2-SR slightly sinuate; r:3-SR:SR1 = 1:5:8; vein SR1 slightly curved upward; vein m-cu distinctly postfurcal and sublinear with vein 2-M; second submarginal cell relatively long (Fig. 49); first subdiscal cell closed; vein CU1b present. Hind wing: vein 1r-m 0.7× as long as vein 1-M; vein m-cu short, oblique, pigmented and straight; vein 2-M pigmented.

**Figure 48.**
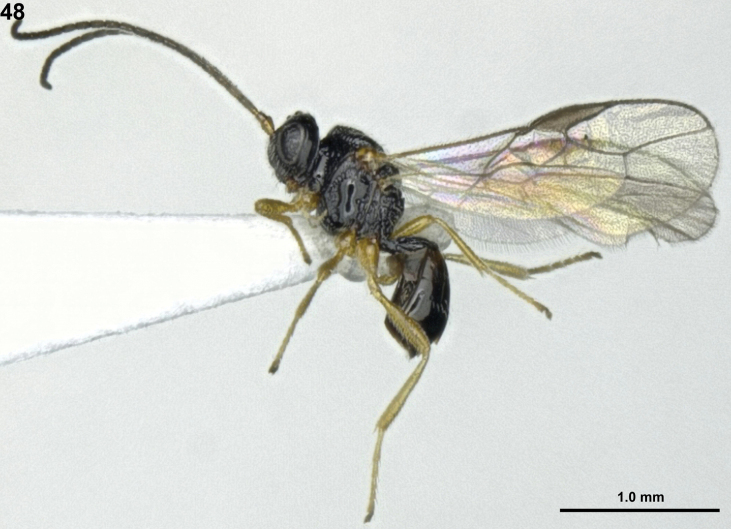
*Sternaulopiusmacrophthalmos* Sheng & Chen, ♂, Japan, habitus, lateral.

***Legs*.** Length of hind femur 4.2× its maximum width (Fig. 48).

***Metasoma*.** First metasomal tergite 1.3× longer than its apical width, its surface rugose with striae, convex medially in lateral view (Fig. 51); dorsope present (Figs 50, 51); second tergite shiny and smooth, with pair of narrow depressions basally; third tergite convex posteriorly in lateral view; following tergites shiny and smooth, with band or row of setae posteriorly.

***Colour*.** Body generally black (Fig. 48); socket of antenna, mandible, tegulae and legs, brownish-yellow; palpi, light yellow; basal part of second metasomal tergite, brown; antenna, pterostigma and veins of wings, dark brown; wings, hyaline.

#### Distribution.

Japan (new record), China (Sichuan and Jilin).

#### Biology.

Unknown.

#### Remarks.

This species runs to *Sternaulopius* Fischer in the key by Sheng et al. (2019), specifically to *S.macrophthalmos*, but it differs by having the mesoscutum more setose and less shiny than in the holotype of *S.macrophthalmos*, the somewhat smaller medio-posterior depression of the mesoscutum and less distinct posterior part of notauli, length of eye 2.2× temple in dorsal view (2.8× longer than temple according to description but 2.6× in fig. 34 of the original description), the less curved vein 1-M of fore wing, hind femur 4.2× longer than its width (4.8× longer than its width according to description but 4.3× in fig. 36 of the original description) and second tergite and following tergites shiny and smooth with band or row of setae posteriorly (without distinct band or row of setae). The holotype of *S.macrophthalmos* was collected in alcohol in a Malaise trap and later treated by the AXA method (specimens were chemically treated with a mixture of xylene + alcohol 96% and amyl acetate, respectively (van Achterberg 2009; van Achterberg et al. 2010). The collecting method and the chemical treatment explain the cleanness of specimen, as well as the shinier appearance and loss of dorsal setae. The relative size of the eyes and legs may be related to the difference in sex (the holotype is female); the other differences are not enough to assign the specimen from Japan to a separate species.

**Figures 49–57.**
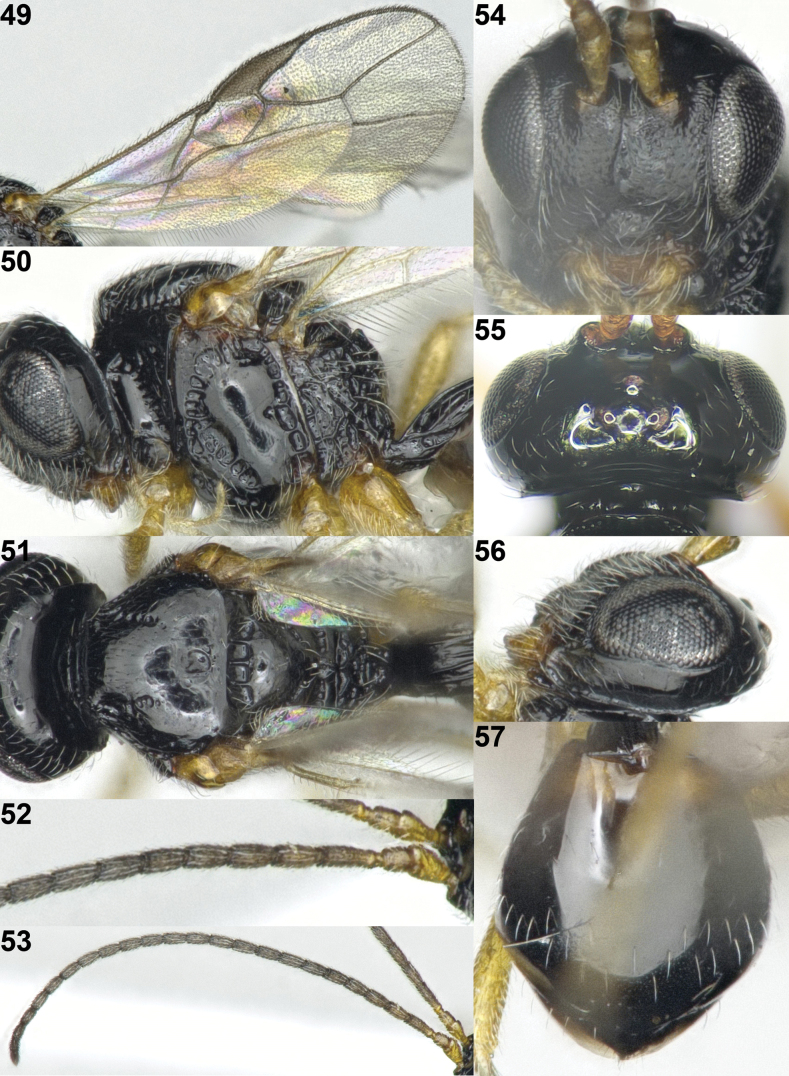
*Sternaulopiusmacrophthalmos* Sheng & Chen, ♂, Japan. **49** wings **50** mesosoma lateral **51** mesosoma dorsal **52** base of antenna **53** antenna **54** head anterior **55** head dorsal **56** head lateral **57** metasoma dorsal.

## Supplementary Material

XML Treatment for Areotetes

XML Treatment for Areotetes convergens

XML Treatment for Indiopius

XML Treatment for Indiopius chenae

XML Treatment for Neopius

XML Treatment for Neopius citrinus

XML Treatment for Sternaulopius

XML Treatment for Sternaulopius maculiferus

XML Treatment for Sternaulopius macrophthalmos

## ﻿Additional information

### Author ORCIDs

Yunjong Han https://orcid.org/0000-0003-2757-7785

Cornelis van Achterberg https://orcid.org/0000-0002-6495-4853

Hyojoong Kim https://orcid.org/0000-0002-1706-2991

### Data availability

All of the data that support the findings of this study are available in the main text.
